# Phytoalexins of the Pyrinae: Biphenyls and dibenzofurans

**DOI:** 10.3762/bjoc.8.68

**Published:** 2012-04-20

**Authors:** Cornelia Chizzali, Ludger Beerhues

**Affiliations:** 1 Institut für Pharmazeutische Biologie, Technische Universität Braunschweig, Mendelssohnstr. 1, 38106 Braunschweig, Germany

**Keywords:** biphenyls, dibenzofurans, phytoalexins, Pyrinae, *Sorbus aucuparia*

## Abstract

Biphenyls and dibenzofurans are the phytoalexins of the Pyrinae, a subtribe of the plant family Rosaceae. The Pyrinae correspond to the long-recognized Maloideae. Economically valuable species of the Pyrinae are apples and pears. Biphenyls and dibenzofurans are formed de novo in response to infection by bacterial and fungal pathogens. The inducible defense compounds were also produced in cell suspension cultures after treatment with biotic and abiotic elicitors. The antimicrobial activity of the phytoalexins was demonstrated. To date, 10 biphenyls and 17 dibenzofurans were isolated from 14 of the 30 Pyrinae genera. The most widely distributed compounds are the biphenyl aucuparin and the dibenzofuran γ-cotonefuran. The biosynthesis of the two classes of defense compounds is not well understood, despite the importance of the fruit crops. More recent studies have revealed simultaneous accumulation of biphenyls and dibenzofurans, suggesting sequential, rather than the previously proposed parallel, biosynthetic pathways. Elicitor-treated cell cultures of *Sorbus aucuparia* served as a model system for studying phytoalexin metabolism. The key enzyme that forms the carbon skeleton is biphenyl synthase. The starter substrate for this type-III polyketide synthase is benzoyl-CoA. In apples, biphenyl synthase is encoded by a gene family, members of which are differentially regulated. Metabolism of the phytoalexins may provide new tools for designing disease control strategies for fruit trees of the Pyrinae subtribe.

## Review

### 

#### Diversity of biphenyl and dibenzofuran phytoalexins

**Figure 1:**
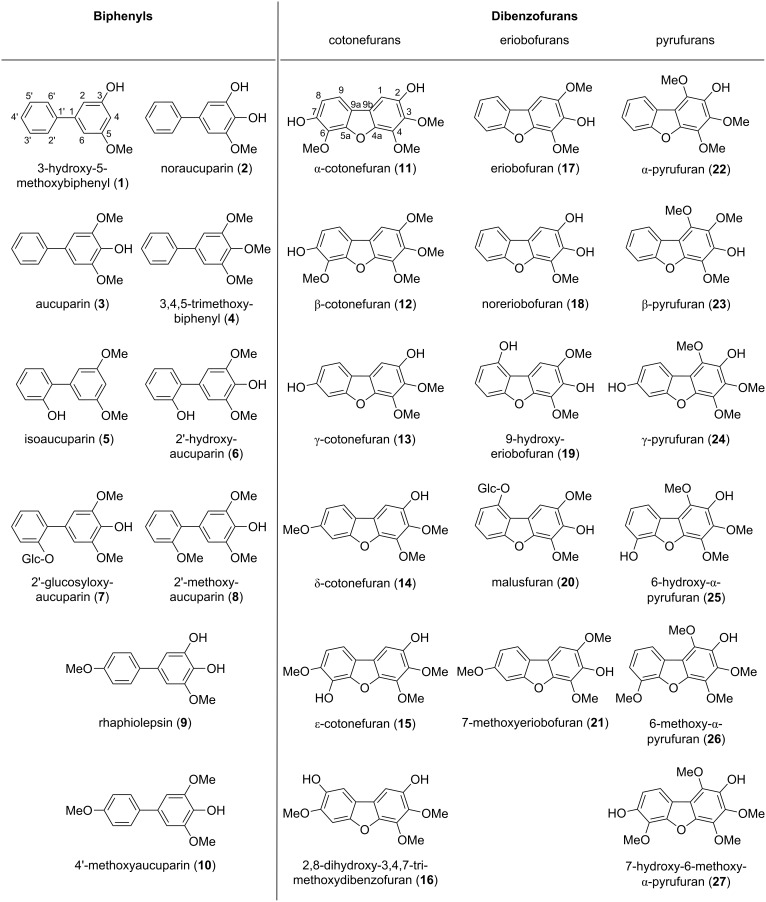
Biphenyl and dibenzofuran phytoalexins isolated from the Pyrinae.

Within the plant family Rosaceae, the subtribe Pyrinae consists of 30 genera and approximately 1000 species, which include a number of economically important fruit trees, such as apple (*Malus domestica*) and pear (*Pyrus communis*) [1]. The subtribe Pyrinae corresponds to the long-recognized subfamily Maloideae, in which the fruit type is generally a pome. In response to biotic and abiotic stress factors, the Pyrinae produce biphenyls and dibenzofurans as phytoalexins, i.e., de novo formed antimicrobial compounds [2]. To date, 10 biphenyls and 17 dibenzofurans have been detected in 14 genera of the Pyrinae (Figure 1) [3–23]. The majority of these inducible defense compounds were found as a result of fungal attack. Six biphenyls (**3**, **5**, **6**, **8**–**10**) and 15 dibenzofurans (**11**–**17**, **19**, **21**–**27**) accumulated in Pyrinae plants after either natural infection or artificial inoculation [3–9,13–19,21]. A single publication reports biphenyl and dibenzofuran formation in response to bacterial challenge [12]. Inoculation of an apple cultivar with the fire-blight-causing bacterium, *Erwinia amylovora*, led to accumulation of four biphenyls (**1**–**3**, **6**) and two dibenzofurans (**17**, **18**). In a fire-blight-infected pear cultivar, three biphenyls (**3**, **4**, **6**) and one dibenzofuran (**18**) were formed. When copper as an abiotic elicitor was applied to leaves of 130 Rosaceae species, including 34 species of the Pyrinae, only *Sorbus aucuparia* formed a phytoalexin, namely aucuparin (**3**) [20]. Another abiotic elicitor, mercury, caused accumulation of 4'-methoxyaucuparin (**10**) in *Rhaphiolepis umbellata* at concentrations higher than after fungal infection [14,19]. So far, no glycosides of biphenyls and dibenzofurans were detected in intact plants of the Pyrinae; however, cell cultures of an apple cultivar accumulated the biphenyl derivative 2'-glucosyloxyaucuparin (**7**) and the dibenzofuran glucoside malusfuran (**20**) [10–11].

The most widely distributed biphenyl is aucuparin (**3**), which was detected as a defense compound in eight Pyrinae species belonging to the six genera *Aronia*, *Chaenomeles*, *Eriobotrya*, *Malus*, *Pyrus*, and *Sorbus*. In contrast, there are biphenyl phytoalexins that are unique to a single species, such as 3-hydroxy-5-methoxybiphenyl (**1**) and 2'-glucosyloxyaucuparin (**7**) in *M. domestica* [11–12], 3,4,5-trimethoxybiphenyl (**4**) in *P. communis* [12], rhaphiolepsin (**9**) in *R. umbellata* [18], and isoaucuparin (**5**) in *S. aucuparia* [21]. Similar observations were made with dibenzofurans. γ-Cotonefuran (**13**) was found as phytoalexin in 11 species of the four genera *Cotoneaster*, *Crataegus*, *Pyrus*, and *Sorbus* [3–4]. In contrast, the following dibenzofurans were detected only in one Pyrinae species: Malusfuran (**20**) in *M. domestica* [10], 7-methoxyeriobofuran (**21**) in *Photinia davidiana* [3], 9-hydroxyeriobofuran (**19**) in *Pyracantha coccinea* [3], α-, β-, and γ-pyrufurans (**22**–**24**) in *P. communis* [3,16–17], and 6-hydroxy-α-pyrufuran (**25**), 6-methoxy-α-pyrufuran (**26**), and 7-hydroxy-6-methoxy-α-pyrufuran (**27**) in *Mespilus germanica* [13].

The number of biphenyl and dibenzofuran phytoalexins strongly varies between the Pyrinae species (Table 1 and Table 2). Some species produced a remarkable array of compounds, whereas others accumulated only a single phytoalexin. For example, five dibenzofurans (**11**–**15**) were observed in *Cotoneaster acutifolius* [3–4], whereas a single dibenzofuran was detected in *C. lactea* (**11**) and *C. veitchii* (**13**) [3,5]*.* Six genera (*Cotoneaster*, *Crataegus*, *Cydonia*, *Mespilus*, *Pseudocydonia*, and *Pyracantha*) lack biphenyls but contain dibenzofurans [3–5,13,15]. Conversely, three genera (*Aronia*, *Chaenomeles*, and *Rhaphiolepis*) lack dibenzofurans but contain biphenyls [3,18–19]. In 16 of the 30 Pyrinae genera, neither biphenyls nor dibenzofurans were detected.

**Table 1:** Occurrence of biphenyl phytoalexins in species of the Pyrinae.

Species	Biphenyls^a^										Reference
	**1**	**2**	**3**	**4**	**5**	**6**	**7**	**8**	**9**	**10**	

***Aronia*** *A. arbutifolia*			+					+		+	[3]
***Chaenomeles*** *C. cathayensis*			+					+		+	[3]
*C. japonica*								+		+	[3]
***Eriobotrya*** *E. japonica*			+								[6–7]
***Malus*** *M. domestica*	+	+	+			+	+	+			[3,9,11–12]
*M. sieversii*			+					+			[3]
*M. silvestris*			+					+		+	[3]
***Photinia*** *P. glabra*								+		+	[14]
***Pyrus*** *P. communis*			+	+		+					[12]
***Rhaphiolepis*** *R. umbellata*									+	+	[18–19]
***Sorbus*** *S. aucuparia*		+	+		+	+		+		+	[3,20–23]

^a^**1**: 3-hydroxy-5-methoxybiphenyl, **2**: noraucuparin, **3**: aucuparin, **4**: 3,4,5-trimethoxybiphenyl, **5**: isoaucuparin, **6**: 2'-hydroxyaucuparin, **7**: 2'-glucosyloxyaucuparin, **8**: 2'-methoxyaucuparin, **9**: rhaphiolepsin, **10**: 4'-methoxyaucuparin.

**Table 2:** Occurrence of dibenzofuran phytoalexins in species of the Pyrinae.

Species	Dibenzofurans^a^																	Reference
	**11**	**12**	**13**	**14**	**15**	**16**	**17**	**18**	**19**	**20**	**21**	**22**	**23**	**24**	**25**	**26**	**27**	

***Cotoneaster*** *C. acutifolius*	+	+	+	+	+													[3–4]
*C. divaricatus*	+	+	+															[3]
*C. henryanus*	+	+	+															[3]
*C. horizentalis*	+	+	+		+													[3]
*C. lactea*	+																	[3,5]
*C. splendens*	+		+															[3]
*C. veitchii*			+															[3]
***Crataegus*** *C. monogyna*	+		+															[3]
***Cydonia*** *C. oblonga*				+	+	+												[3]
***Eriobotrya*** *E. japonica*							+											[8]
***Malus*** *M. domestica*							+	+		+								[10,12]
***Mespilus*** *M. germanica*	+														+	+	+	[3–5,13]
***Photinia*** *P. davidiana*							+				+							[3,15]
***Pseudocydonia*** *P. sinensis*					+													[3]
***Pyracantha*** *P. coccinea*							+		+									[3,15]
***Pyrus*** *P. communis*						+		+				+	+	+				[3,16–17]
*P. nivalis*			+			+												[3]
*P. ussuriensis*			+			+												[3]
*P. pyraster*						+												[3]
***Sorbus*** *S. aucuparia*							+	+										[23]
*S. chamaemespilus*			+															[3]
*S. domestica*			+															[3]

^a^**11**: α-cotonefuran, **12**: β-cotonefuran, **13**: γ-cotonefuran, **14**: δ-cotonefuran, **15**: ε-cotonefuran, **16**: 2,8-dihydroxy-3,4,7-trimethoxydibenzofuran, **17**: eriobofuran, **18**: noreriobofuran, **19**: 9-hydroxyeriobofuran, **20**: malusfuran, **21**: 7-methoxyeriobofuran, **22**: α-pyrufuran, **23**: β-pyrufuran, **24**: γ-pyrufuran, **25**: 6-hydroxy-α-pyrufuran, **26**: 6-methoxy-α-pyrufuran, **27**: 7-hydroxy-6-methoxy-α-pyrufuran.

Outside the subtribe Pyrinae, biphenyls and dibenzofurans were also found in a number of species. However, they do not function as phytoalexins, i.e., de novo formed defense compounds after microbial infection. They occur as preformed constituents (phytoanticipins), which are present before any challenge by microorganisms or herbivores and provide a constitutive barrier.

#### Antimicrobial properties

Antifungal activity of biphenyls and dibenzofurans was demonstrated in a number of studies [4,7–10,12–15,18–19,21]. Spore germination, germ-tube development, and mycelial growth were inhibited by the phytoalexins at concentrations that are supposed to be present at local infection sites [10]. For example, the effective dose 50% (ED_50_) for inhibition of *Fusarium culmorum* ranged from 12 to 84 μg/mL [4,13,15,21]. When the dibenzofuran eriobofuran (**17**) and its *O*-glucoside malusfuran (**20**) were tested for their inhibitory effect on the scab-causing fungus, *Venturia inaequalis*, the aglycone exhibited significantly stronger antifungal activity than the glucoside [10]. This finding agrees with the observation that the accumulated phytoalexins are commonly aglycones of biphenyls and dibenzofurans.

The antibacterial activity of biphenyls and dibenzofurans is less well studied [24–25]. Recently, a number of the Pyrinae-specific phytoalexins were tested for in vitro antibacterial activity against *E. amylovora*, the fire-blight-causing agent [12]. 3,5-Dihydroxybiphenyl was the most active compound with a minimum inhibitory concentration (MIC) of 115 μg/mL. While this concentration was bactericidal, a concentration approximately ten times lower led to 50% growth inhibition (MIC_50_ = 17 μg/mL). Biphenyls exhibited somewhat stronger antibacterial activity than structurally related dibenzofurans did [12], whereas the opposite tendency was observed for antifungal activity [26]. However, more biphenyls and dibenzofurans need to be tested for their antibacterial and antifungal potentials in order to allow for reliable conclusions concerning structure–activity relationships. The array of phytoalexins accumulated in response to infection in a number of Pyrinae species appears to provide protection from both bacterial and fungal pathogens, such as *E. amylovora* and *V. inaequalis*, respectively. The mechanism of antimicrobial action of biphenyls and dibenzofurans has not yet been established.

#### Co-occurrence of biphenyls and dibenzofurans

In a previous study [3], it was concluded that Pyrinae species produce either biphenyls or dibenzofurans. No plant was known to simultaneously produce both classes of phytoalexins. *Malus* was a biphenyl producer and *Pyrus* was a dibenzofuran producer. *Eriobotrya japonica* was found to form the biphenyl aucuparin (**3**) and the dibenzofuran eriobofuran (**17**); however, the former compound was present in the cortex and the latter in the leaves [6–8]. Generally, species of the same genus produce the same class of phytoalexins, except for *Photinia glabra*, which contained biphenyls (**8**, **10**), and *P. davidiana*, which formed dibenzofurans (**17**, **21**) [3,14–15]. Based on the lack of co-occurrence of biphenyl and dibenzofuran phytoalexins, parallel, rather than sequential, biosynthetic pathways were proposed [3]. Later, the simultaneous formation of biphenyls and dibenzofurans was observed in elicitor-treated cell cultures of a scab-resistant apple cultivar, which formed the biphenyls aucuparin (**3**), 2'-hydroxyaucuparin (**6**), and 2'-glucosyloxyaucuparin (**7**) in addition to the dibenzofuran malusfuran (**20**) [10–11]. For intact plants, co-occurrence of the two classes of defense compounds has only recently been observed in fire-blight-infected stems of apple and pear [12]. While the pear cultivar accumulated three biphenyls (**3**, **4**, **6**) and one dibenzofuran (**18**), the apple cultivar formed four biphenyls (**1**–**3**, **6**) and two dibenzofurans (**17**, **18**) [12]. Along with the previously isolated compounds, apple species produce seven biphenyls (**1**–**3**, **6**–**8**, **10**) and three dibenzofurans (**17**, **18**, **20**) [3,9–12], whereas pear species form three biphenyls (**3**, **4**, **6**) and six dibenzofurans (**13**, **16**, **18**, **22**–**24**) [3,12,16–17].

#### Elicitor-treated cell cultures as a model system

Cell suspension cultures treated with elicitors are widely used to investigate microbe-induced processes in systems of reduced complexity, as compared to natural interactions between differentiated plants and intact pathogens [22,27–32]. The phytoalexin response in elicitor-treated cell cultures is magnified relative to that at local infection sites of plant organs. Furthermore, the disruption of cultured cells to extract phytoalexins, as well as enzymes and transcripts, is easier than homogenization of intact, woody plants. However, cell cultures fail to provide insight into the organ and tissue specificities of the biosynthetic pathway.

**Figure 2:**
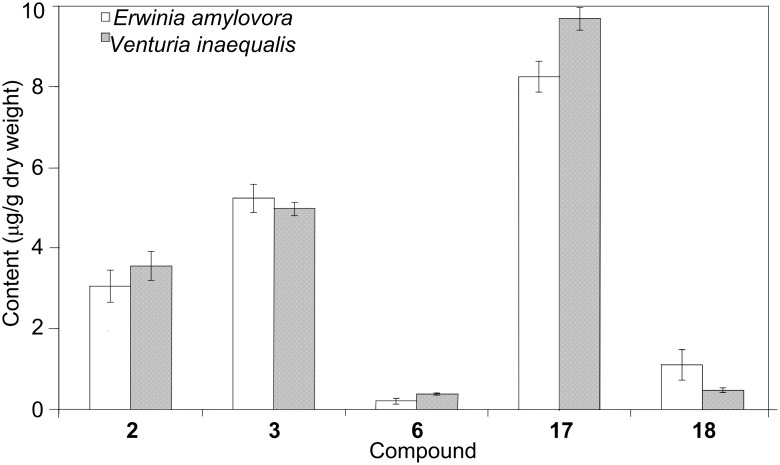
Biphenyl and dibenzofuran concentrations determined in *S. aucuparia* cell cultures after treatment with *E. amylovora* and *V. inaequalis* [23]. Data are average values ± SD (*n* = 3).

**Figure 3:**
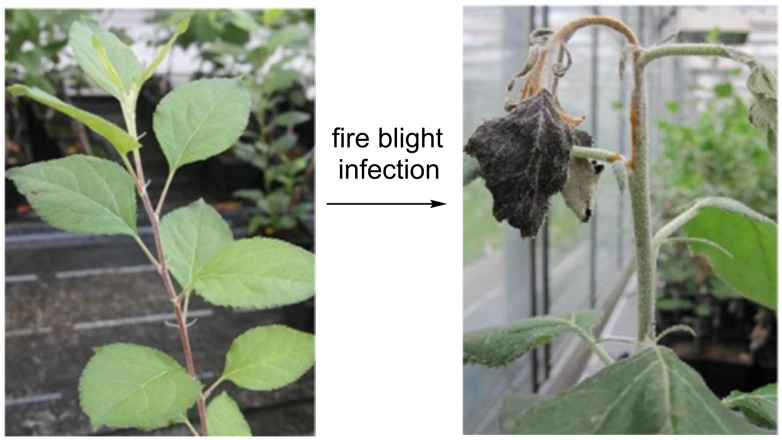
Greenhouse-grown apple shoots inoculated with the fire-blight-causing bacterium, *E. amylovora*.

We have established cell cultures of *S. aucuparia* as a model system for studying biphenyl and dibenzofuran formation after elicitor treatment [22–23]. *S. aucuparia* cell cultures respond to the addition of elicitors with the accumulation of both biphenyls (**2**, **3**, **6**) and dibenzofurans (**17**, **18**). Simultaneous formation of the two classes of defense compounds has thus been observed with *M. domestica*, *P. communis*, and *S. aucuparia*, although intact *S. aucuparia* plants contain only biphenyls [3,20–22]. The pattern of phytoalexins formed in *S. aucuparia* cell cultures varied with the type of elicitor added [23]. Yeast extract mainly induced the formation of aucuparin (**3**), whereas chitosan, although being a relatively poor elicitor, primarily stimulated the production of noraucuparin (**2**). Maximum phytoalexin levels were observed after the addition of autoclaved suspensions of the fire-blight bacterium, *E. amylovora*, and the scab-causing fungus, *V. inaequalis*. Eriobofuran (**17**) was the major inducible defense compound. The total biphenyl and dibenzofuran concentrations were 8.5 and 9.5 μg/g dry weight, respectively, and did not appreciably differ after treatment with the scab fungus and the fire-blight bacterium (Figure 2). These two pathogens along with the powdery mildew-causing fungus are responsible for the most destructive diseases affecting the Pyrinae, which lead to significant yield losses and crop failures in apple and pear production (Figure 3) [33].

#### Biosynthesis of biphenyls and dibenzofurans

**Figure 4:**
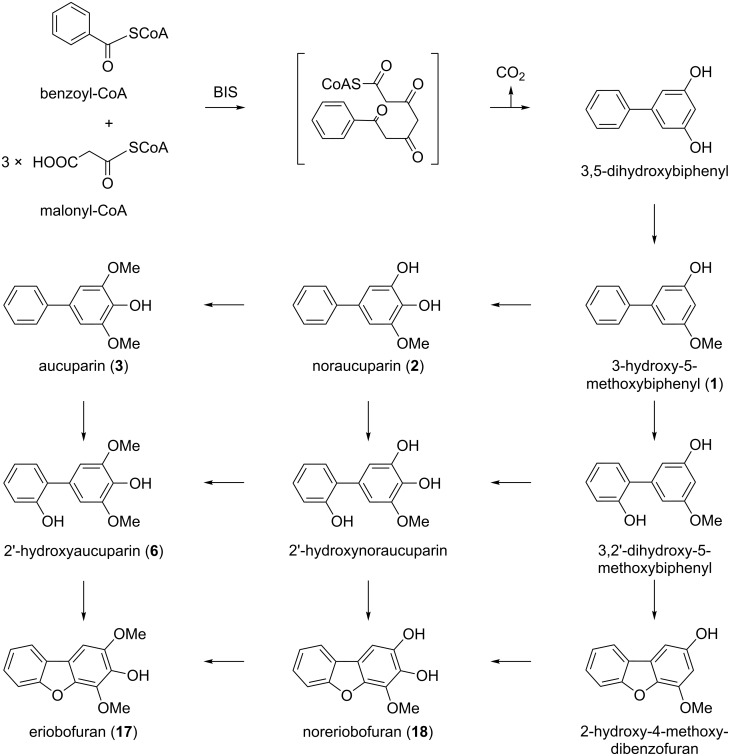
Established formation of 3,5-dihydroxybiphenyl by biphenyl synthase (BIS) [34] and proposed biosynthetic reactions leading to biphenyl and dibenzofuran phytoalexins in elicitor-treated *S. aucuparia* cell cultures [23].

The key enzyme of the biosynthetic pathway is biphenyl synthase (BIS) [22]. This type-III polyketide synthase (PKS) catalyzes the iterative condensation of benzoyl-CoA with three acetyl units from the decarboxylation of malonyl-CoA to form a linear tetraketide intermediate, which undergoes intramolecular C2→C7 aldol condensation and decarboxylative elimination of the terminal carboxyl group to give 3,5-dihydroxybiphenyl (Figure 4). BIS activity was first detected in cell cultures of *S. aucuparia* treated with yeast extract as an elicitor [22]. A BIS cDNA was cloned, and the recombinant enzyme was functionally expressed in *Escherichia coli* and characterized [34]. Recently, four cDNAs encoding BIS isoenzymes were cloned from fire-blight-infected shoots of apple plants, heterologously expressed, and functionally analyzed [35]. Expression of the four BIS genes was differentially regulated in response to fire-blight infection. While the BIS3 gene was expressed in stems, leading to the accumulation of four biphenyls (**1**–**3**, **6**) and two dibenzofurans (**17**, **18**), the BIS2 gene was transcribed in leaves. However, leaves failed to accumulate immunodetectable amounts of BIS protein, which was consistent with the absence of phytoalexins from the leaves [35]. In cell cultures of apple, three BIS genes were expressed after treatment with an autoclaved suspension of the fire-blight bacterium. Immunofluorescence localization in cross sections of apple stems revealed the occurrence of the BIS protein in the parenchyma of the bark [35]. Interestingly, dot-shaped immunofluorescence was confined to the junctions between neighboring cortical parenchyma cells, suggesting an association of BIS with plasmodesmata.

**Figure 5:**
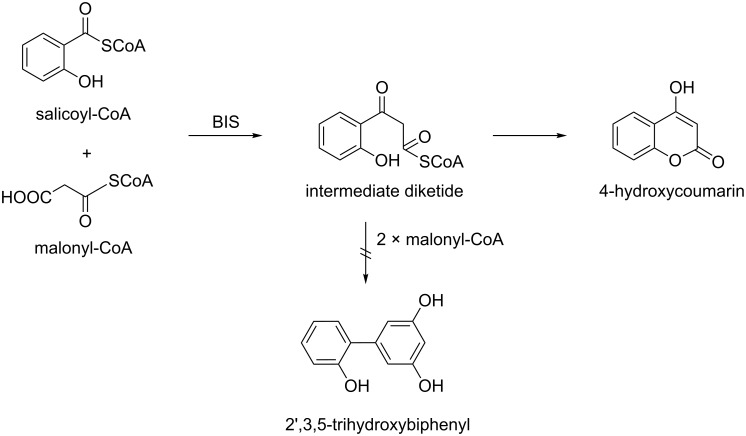
In vitro biosynthesis of 4-hydroxycoumarin by biphenyl synthase (BIS). No formation of 2',3,5-trihydroxybiphenyl was observed [35–36].

The simultaneous formation of biphenyls and dibenzofurans in *M. domestica* [3,9–12], *P. communis* [3,12,16–17], and *S. aucuparia* [3,20–23] led us to propose sequential, rather than parallel, pathways of biphenyl and dibenzofuran biosynthesis (Figure 4) [23]. BIS thus appears to form the carbon skeleton of both classes of defense compounds. The product of the BIS reaction, 3,5-dihydroxybiphenyl, undergoes O-methylation to give 3-hydroxy-5-methoxybiphenyl (**1**), as recently detected in elicitor-treated *S. aucuparia* cell cultures (Khalil and Beerhues, unpublished). Subsequent 4-hydroxylation and additional O-methylation yield noraucuparin (**2**) and aucuparin (**3**), respectively. The dibenzofurans noreriobofuran (**18**) and eriobofuran (**17**) have been proposed to arise from 2'-hydroxylated intermediates, one of which, 2'-hydroxyaucuparin (**6**), was isolated from *S. aucuparia* cell cultures [23]. Interestingly, the 2'-hydroxylated intermediates do not originate from salicoyl-CoA as a starter substrate [35–36]. All BIS enzymes studied so far released 4-hydroxycoumarin after a single condensation with malonyl-CoA rather than 2',3,5-trihydroxybiphenyl after three additions of acetyl units (Figure 5). Intramolecular cyclization converting 2'-hydroxylated intermediates to dibenzofurans has not yet been demonstrated biochemically (Figure 4).

## Conclusion

Upon attack by pathogens, species of the Pyrinae form biphenyl and dibenzofuran phytoalexins. The biosynthesis of these two classes of defense compounds is poorly understood, although the Pyrinae include apple, pear, and related fruit trees. Plant diseases, such as fire blight, scab, and powdery mildew, lead to dramatic losses of fruits and trees. Engineering of the phytoalexin metabolism may provide new tools for enhancing disease resistance in economically important cultivars. However, this approach requires a detailed knowledge of biphenyl and dibenzofuran biosynthesis at the metabolic, enzymatic, and genetic levels. Data obtained with elicitor-treated cell cultures as a simplified experimental system lay the foundation for the study of the more complex interaction of differentiated plants and intact pathogens.
